# Impact of Post-Exercise Fructose-Maltodextrin Ingestion on Subsequent Endurance Performance

**DOI:** 10.3389/fnut.2020.00082

**Published:** 2020-06-05

**Authors:** Tim Podlogar, Gareth A. Wallis

**Affiliations:** School of Sport, Exercise and Rehabilitation Sciences, College of Life and Environmental Sciences, University of Birmingham, Birmingham, United Kingdom

**Keywords:** nutrition, sport, sugars, fatigue, metabolism, recovery

## Abstract

**Background:** Current sports nutrition guidelines recommend athletes ingest carbohydrates at 1.0–1.2 g·kg^−1^·h^−1^ to optimize repletion of muscle glycogen during short-term recovery from endurance exercise. However, they do not provide specific advice on monosaccharides (e.g., fructose or glucose) other than to ingest carbohydrates of moderate to high glycaemic index. Recent evidence suggests that combined ingestion of fructose and glucose in recovery leads to enhanced liver glycogen synthesis and that this translates into improvement of subsequent endurance capacity.

**Purpose:** The purpose of the present study was to investigate whether consuming a combination of fructose and glucose as opposed to glucose alone during short-term recovery (i.e., 4 h) from exhaustive exercise would also improve subsequent pre-loaded cycle time trial (TT) performance.

**Methods:** Eight participants (seven men, one woman; $\overset{∙}{\text{V}}$O_2_peak: 56.8 ± 5.0 mLO_2_·min^−1^·kg^−1^; Wmax: 352 ± 41 W) participated in this randomized double-blind study. Each experimental session involved a glycogen reducing exercise bout in the morning, a 4-h recovery period and 1-h of steady state (SS) exercise at 50% Wmax followed by a ~40-min simulated TT. During recovery carbohydrates were ingested at a rate of 1.2 g·kg^−1^·h^−1^ in the form of fructose and maltodextrin (FRU + MD) or dextrose and maltodextrin (GLU + MD) (both in 1:1.5 ratio). Substrate oxidation rates, including ingested carbohydrate oxidation, were determined during the steady state (SS). Blood samples were collected during recovery, during the SS exercise and at the end of the TT for determination of glucose and lactate concentrations.

**Results:** There were no differences in TT performance [37.41 ± 3.45 (GLU + MD); 37.96 ± 5.20 min (FRU + MD), *p* = 0.547]. During the first 45-min of SS oxidation of ingested carbohydrates was greater in FRU + MD (1.86 ± 0.41 g^−1^·min^−1^ and 1.51 ± 0.37 g^−1^·min^−1^ for FRU + MD and GLU + MD, respectively; time x condition interaction *p* = 0.003) and there was a trend toward higher overall carbohydrate oxidation rates in FRU + MD (2.50 ± 0.36 g^−1^·min^−1^ and 2.31 ± 0.37 g^−1^·min^−1^ for FRU + MD and GLU + MD, respectively; *p* = 0.08). However, at 60-min of SS, differences in substrate oxidation disappeared.

**Conclusion:** Ingestion of combined fructose and glucose compared to glucose only during recovery from an exhaustive exercise bout increased the ingested carbohydrate oxidation rate during subsequent exercise. Under the conditions studied, subsequent TT performance was not improved with fructose-glucose.

## Introduction

Numerous top level athletes undertake multiple training sessions daily (1, 2) and it is common in certain sports that competitive elements follow each other with limited (i.e., a few hours) time for recovery. During prolonged strenuous exercise carbohydrate oxidation accounts for a large proportion of energy expenditure (3) and muscle and liver glycogen content can be substantially reduced (4, 5). From a metabolic perspective, if recovery of the ability to perform strenuous exercise is a priority, it is important to replenish both muscle and liver glycogen stores. Guidelines suggest that in order to maximize short term post-exercise muscle glycogen synthesis moderate to high glycaemic carbohydrates should be ingested as soon as possible at a rate 1.0–1.2 g·kg^−1^·h^−1^ (6). However, evidence suggests that carbohydrate type can also influence liver glycogen synthesis and thereby impact post-exercise recovery (7, 8).

The most recent evidence indicates that combining fructose- and glucose- based carbohydrate sources (e.g., a combination of fructose and maltodextrin, or sucrose) leads to higher total glycogen storage as compared to when only glucose-based carbohydrates are ingested. This is thought to be due to the preferential storage of fructose and glucose as liver and muscle glycogen, respectively (4, 9–13). Recently, co-ingestion of fructose and maltodextrin during a 4-h recovery period enhanced recovery of subsequent endurance running capacity as compared to glucose and maltodextrin (14). This has now been verified in a cycling model (15). A metabolic mechanism was inferred in the study by Maunder et al. (14) through observations of increased exogenous and better maintained total carbohydrate oxidation during the second exercise bout. However, the relatively short duration second exercise bout (i.e., exogenous oxidation could be compared between trials for just 30 min of exercise) challenged assumptions related to retention of the ^13^C label in the body CO_2_ pools when using ^13^C labeled carbohydrates for determination of exogenous carbohydrate oxidation (16). In addition, it was not possible to completely discount a role for gastrointestinal (GI) distress in explaining the differences in subsequent exercise capacity when fructose-glucose or glucose only sources were provided.

The purpose of the present study was to test the hypothesis that combined ingestion of fructose and glucose as opposed to glucose alone during short-term recovery from exhaustive exercise improves subsequent pre-loaded cycle time trial performance. Exercise performance was selected as the primary outcome as some have questioned the external validity of measuring exercise capacity that has been assessed in previous studies by Maunder et al. (14) and Gray et al. (17). Cycling was used as the exercise mode given that GI distress is less prevalent in cycling than in running (18) thus improving the potential to isolate metabolic effects from GI influences. In a parallel trial, breath ^13^CO_2_ appearance from ingested ^13^C labeled bicarbonate was determined as a marker of label retention within the bicarbonate pool during exercise, in order to attribute the metabolic effects of post-exercise carbohydrate feeding with more certainty.

## Materials and Methods

### Participants

Eleven healthy, endurance-trained participants (eight men, three women) accustomed to cycling exercise, provided written informed consent and met the eligibility criteria to undertake the study that was approved by the Science, Technology, Engineering and Mathematics Ethics Committee, University of Birmingham, UK (Ethics code: ERN_17-1236). Their mean age, body mass, height, maximal oxygen uptake (V°O_2_peak), and maximal cycle ergometer power output (Wmax) were 31 ± 5 years, 68 ± 9 kg, 173 ± 7 cm, 3.86 ± 0.52 L O_2_·min^−1^ (56.9 ± 4.8 mL O_2_·min^−1^·kg^−1^), and 338 ± 47 W (5.0 ± 0.5 W·kg^−1^), respectively. These participants all completed preliminary testing and their data are included for estimation of ^13^C bicarbonate elimination (further details below). Three participants dropped out after the preliminary or familiarization visits: two due to time commitments; one due to GI discomfort during the familiarization trial. Thus eight participants successfully finished all trials. Their mean age, body mass, height, maximal oxygen uptake (V°O_2_peak), and maximal cycle ergometer power output (Wmax) were 31 ± 5 years, 71 ± 7 kg, 175 ± 8 cm, 4.04 ± 0.44 L O_2_·min^−1^ (56.8 ± 5.0 mL O_2_·min^−1^·kg^−1^), and 352 ± 41 W (5.0 ± 0.5 W·kg^−1^), respectively.

### Experimental Design

Participants underwent preliminary testing that consisted of a V°O_2_peak test and a steady state exercise bout. The latter was used to estimate elimination rate of ingested ^13^C bicarbonate. Participants then completed a familiarization trial and two experimental trials consisting of a glycogen-reducing exercise bout in the morning followed by a 4-h recovery period and a 1-h steady state exercise bout (SS) immediately followed by a time trial (TT) with a predicted duration of 40-min. The experimental trials differed in the diet provided during the 4-h recovery window. On both occasions participants received carbohydrates at a rate of 1.2 g·kg^−1^·h^−1^ during a 4-h re-feeding period with a difference in the composition of carbohydrates. On one occasion they received a mixture of glucose-based carbohydrates, namely dextrose and maltodextrin (GLU + MD) and on another fructose and maltodextrin (FRU + MD), both in a 1:1.5 ratio. The order of the trials was randomized, the study was double-blinded, and the trials were separated by at least 6 days.

### Preliminary Testing and Determination of Bicarbonate Elimination

Participants came to the laboratory at ~07:00 h after an overnight fast. They performed an incremental test to exhaustion to determine V°O_2_peak and Wmax on a cycle ergometer (Excalibur Sport; Lode, Groningen, Netherlands). The test started at a power output of 100 W and the workload increased by 30 W every 2 min. During the test, gas exchange measurements were made using an automated online gas analysis system (Vyntus, Vyaire Medical, Ottawa, IL, US). Gas analyzers were calibrated with a known gas mixture (15.04% O_2_, 5.06% CO_2_; BOC Gases, Surrey, UK) and the volume transducer was calibrated with a 3-liter calibration syringe (Jaeger, Wurzburg, Germany) prior to each trial. The highest 30-s average of O_2_ uptake was considered to represent V°O_2_peak. Wmax was calculated as the power output from the last completed stage plus the fraction of the time spent in the next stage multiplied by 30 W.

Participants then rested for 30-min before undertaking an exercise session assessing kinetics of ^13^C labeled bicarbonate elimination as previously described (19). Stable isotope methodology is commonly used to assess oxidation rates of ingested carbohydrates. However, after their oxidation, labeled carbon atoms previously constituting carbohydrate molecules can be for some time held within the body pool of bicarbonate and thus oxidation rates of ingested carbohydrates underestimated. Thus, it is common to calculate the time required for the bicarbonate pool to be turned over. Briefly, immediately after ingestion of 0.23 ± 0.08 mg · kg body weight of ^13^C labeled bicarbonate (99% purity, Cambridge Isotope Laboratories, Inc.; Andover, USA) participants exercised at 50% Wmax for 60 min. ^13^CO_2_ production, reflecting ^13^C labeled bicarbonate excretion was determined throughout the exercise by measurement of CO_2_ production (as described above using indirect calorimetry) and by quantification of ^13^CO_2_/^12^CO_2_ ratio in expired breath using isotope ratio mass spectrometry (IsoAnalytical Ltd., Crewe, UK). Breath was collected into 10-mL evacuated tubes (Exetainer Breath Vial, Labco Ltd.; Buckinghamshire, UK). Cumulative excretion of labeled bicarbonate was quantified at 2, 5, 10, 20, 30, 45, and 60 min.

### Familiarization and Experimental Trials

A familiarization trial was performed ~7 days after preliminary testing. It followed the same protocol as the experimental trials in GLU + MD condition without blood sampling. As well as familiarization, this trial was used to quantify any background shift in ^13^CO_2_ production during the SS exercise period to enable a more accurate determination of ingested carbohydrate oxidation rates (further described below).

Participants entered the laboratory at ~7:00 after not eating from 22:00 the day before. They were asked to replicate the diet and activity patterns of the day preceding each experimental trial. On entering the laboratory a blood sample was taken using venepuncture from an antecubital vein. Then they performed a high-intensity-interval exercise protocol as described previously (13, 20). This protocol has previously been shown to effectively reduce muscle glycogen concentration (13). After a 5-min warm-up at 50% Wmax participants cycled at alternating workloads of 90 and 50% Wmax, respectively, each lasting 2 min. Once 90% workload was deemed too demanding for participants to be able to cycle at a cadence of more than 60 revolutions per minute (RPM) despite strong verbal encouragement, 90% intensity was first reduced to 80% and then to 70%. When blocks at 70% Wmax could not be completed at the cadence >60 RPM, the exercise session was terminated. Immediately post-exercise an indwelling cannula was placed in an antecubital arm vein and a blood sample taken. Participants then started the 4-h recovery period.

During recovery, participants passively rested for 4 h, during which time sedentary activities such as reading and use of laptops were permitted. Immediately upon obtainment of the blood sample post exercise, participants commenced ingesting a 15% carbohydrate containing drink every 30 min with a carbohydrate ingestion rate of 1.2 g·kg^−1^·h^−1^. Composition of the drink was either glucose (Roquette, Lestrem, France or Myprotein, The Hut Group, Cheshire, UK for familiarization and experimental trials, respectively) and maltodextrin (Avebe, Veendam, The Netherlands or Myprotein, The Hut Group, Cheshire, UK for familiarization and experimental trials, respectively) (GLU + MD) or fructose (Peak Supps, Bridgend, UK) and maltodextrin (FRU + MD) in a 1:1.5 ratio. Carbohydrates ingested during recovery of the familiarization trial were of naturally low ^13^C abundance [−26.17δ ^13^C_V−PDB_ (%0)] whereas during the experimental trials they were naturally high ^13^C abundance [−11.18 δ ^13^C_V−PDB_ (%0) for FRU + MD and −11.14 δ ^13^C_V−PDB_ (%0) for GLU + MD]. A venous blood sample was obtained every 1 h during the recovery with the cannula flushed with saline every 30 min to maintain patency.

During the 1-h long SS at 50% Wmax, participants breathed for 3 min every 15 min into a mouthpiece connected to the metabolic cart (as described above) for the determination of V°O_2_ and V°CO_2_ and breath samples were collected into Exetainers as described above for subsequent mass spectroscopy analysis of ^13^C enrichment in the expired breath. In addition to that, blood samples were collected every 15 min during the SS and at the end of the TT. Immediately on completion of the SS, the cycle ergometer was set to a linear mode (workload increases linearly with the cycling cadence) and the TT started. Participants had to perform a set amount of work (equal to ~40 min of cycling at 65% W_max_) as quickly as possible. The amount of work for each participant was calculated according to the following equation:

Total amount of work = 0.65 Wmax × 2,400 J

The ergometer was set in the linear mode and the linear factor calculated according to the formula:

$$\begin{array}{l} L=W/{\left(RPM\right)}^{2} \end{array}$$

Where L is a linear factor, W is predicted power and RPM is the cycling cadence. RPM was set to 80, where W represented 65% Wmax. During the TT, participants were displayed the total amount of work to be completed, remaining work and % of work completed, while the cadence and the power output were recorded but not visible to the participants. As per recommendations (17), the TT took place in silence with no verbal encouragement given. Following the TT, a final blood sample was collected.

### Blood Analyses

Venous blood samples (~6 mL) were collected into EDTA tubes, stored on ice and then centrifuged at 4°C and 1,006 × g for 15 min. Aliquots of plasma were then stored at −70°C and later analyzed for glucose (Glucose kit; Randox, London, UK) and lactate (Lactate kit; Randox, London, UK) using an automated photometric based clinical chemistry analyzer RX Daytona+ (Randox, London, UK).

### Gas Exchange Measurements

Fat and carbohydrate oxidation rates were calculated using stoichiometric equations of Jeukendrup and Wallis (21) with the following equations assuming protein oxidation to be negligible:

$$\begin{array}{l} CHO\;Oxidation=4.210\;\overset{∙}{V}CO_{2}-2.962\;\overset{∙}{V}O_{2}\\ Fat\;Oxidation=1.695\;\overset{∙}{V}O_{2}-1.701\;\overset{∙}{V}CO_{2} \end{array}$$

Where $\overset{∙}{\text{V}}$CO_2_ and $\overset{∙}{\text{V}}$O_2_ are expressed in L·min^−1^ and oxidation rates (CHO and fat) are calculated in g·min^−1^.

The isotopic enrichment was expressed as δ per milliliter difference between the ^13^C/^12^C ratio of the sample and a known laboratory reference standard.

The percentage of elimination of ingested ^13^C bicarbonate was calculated according to the following equation (19):

$$\begin{array}{l} {\%}^{13}C_{elimination}=\frac{85\cdot {\overset{∙}{V}}_{{}^{13}CO_{2}}}{0.0224\cdot m_{bicarb}}\cdot 100 \end{array}$$

Where 85 represents molecular weight of ingested sodium bicarbonate, ${\overset{∙}{V}}_{{}^{13}CO_{2}}$ volume of expired ^13^CO_2_ (L), 22.4 L is volume of air occupied by 1 mol of CO_2_ and m_bicarb_ mass (mg) of ingested bicarbonate. ${\overset{∙}{V}}_{{}^{13}CO_{2}}$was calculated by multiplying the volume of expired gas and the atom per cent excess of ^13^CO_2_.

The ingested carbohydrate oxidation rate during SS, representing oxidation of the carbohydrates ingested during recovery, were calculated according to the following equation (22):

$$\begin{array}{l} CHO_{ing}=\overset{∙}{V}CO_{2}\left(\frac{\delta \text{Exp}-{\text{Exp}}_{bkg}}{\delta \text{Ing}-{\text{Exp}}_{bkg}}\right)\cdot \left(\frac{1}{0.07467}\right) \end{array}$$

Where δ Exp represents ^13^C enrichment of expired gas sample, δ Ing represents ^13^C represents enrichment of ingested carbohydrate, Exp_bkg_ represents enrichment of expired gas sampled during the familiarization session at the corresponding timepoint, and 0.7467 $\overset{∙}{\text{V}}$CO_2_ of 1 g glucose oxidation.

### Heart Rate, Rate of Perceived Exertion and Gastrointestinal Comfort

During recovery, gastrointestinal comfort (GC) was assessed every hour using a 10-point Likert scale (23) that included assessment of experience of nausea, stomach fullness and abdominal cramping. Heart rate (HR) values were obtained at 15-min intervals during the SS via a heart rate strap (H7, Polar, Kempele, Finland) which was connected via Bluetooth® to a watch (Ambit 3 Sport, Suunto, Vantaa, Finland). Simultaneously, every 15-min participants were asked to report the rate of perceived exertion (RPE) using 6–20 scale (24) and GC using the same questionnaire as during the recovery.

### Statistics

Sample size was determined using g^*^power software (25) assuming an effect size of 1.84 as observed previously for differences in subsequent exercise capacity between the two carbohydrate conditions (14). It was calculated that to achieve statistical power of 80% to detect differences between the two conditions, a minimum of seven subjects should complete both experimental trials. A two-way ANOVA for repeated measures was used to compare differences in substrate utilization and blood metabolites at different time points. Where significant effects were observed by ANOVA for time x condition interaction, *post-hoc* comparisons were made with paired *t*-tests with the Tukey test applied to account for multiple comparisons. TT performance and amount of work completed during glycogen reducing exercise sessions were initially tested for normality using Shapiro-Wilk test. If normality was met, a *t*-test was used to analyse the data, otherwise the nonparametric Wilcoxon signed-rank test was used. All values are presented as mean ± SD. Statistical significance was set at *p* < 0.05. Data analysis was performed using SPSS (Version 24; SPSS Inc., Chicago, IL, US), Prism (Version 8; GraphPad Software, San Diego, CA, US) and Microsoft Excel (Microsoft, Redmond, Washington, USA).

## Results

### Bicarbonate Elimination

**Figure 1:**
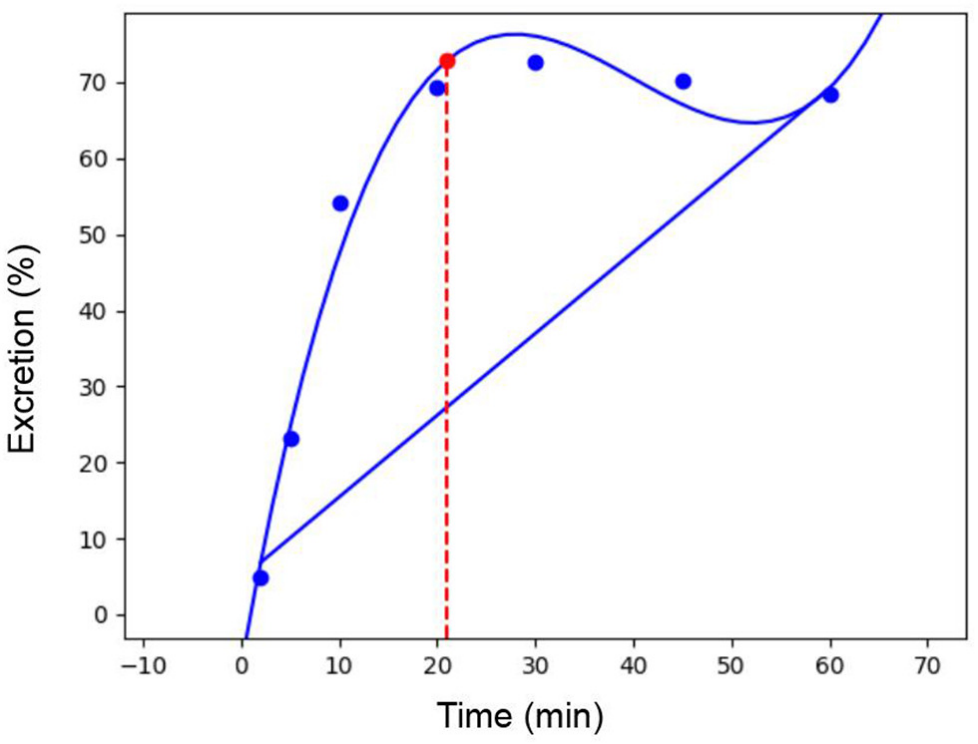
Elimination of ingested ^13^C labeled bicarbonate.

The D-max method (26) was used to determine the point when a plateau in excretion of ^13^C atoms had been reached. This point occurred at 21.04 ± 0.87 min. An example of the calculation for one participant can be seen in Figure 1.

### Glycogen-Reducing Session

Participants completed 1,367 ± 421 and 1,298 ± 571 kJ of work during the glycogen reducing sessions in GLU + MD and FRU + MD, respectively, without any statistically significant differences between the trials (*p* = 0.662). Neither were there any differences in the number of completed stages at 90, 80, and 70% W_max_ between all three conditions (*p* = 0.458).

### TT Performance

**Figure 2:**
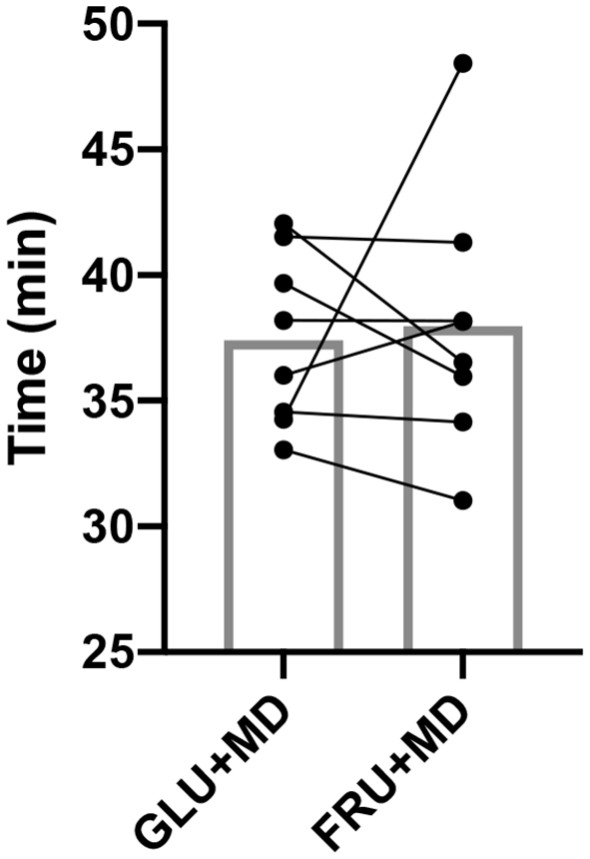
Time to complete the TT. Bars represent mean, while dots and connecting lines represent individual participants.

Results for TT can be seen in Figure 2, Participants finished the TT in 37.41 ± 3.45 (GLU + MD) and 37.96 ± 5.20 min (FRU + MD) without any significant difference between them (*p* = 0.547).

### Carbohydrate and Fat Oxidation Rates, $\overset{∙}{\text{V}}$O_2_, RPE and HR During Steady State Exercise

**Figure 3:**
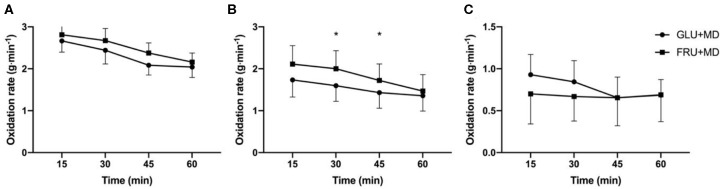
**(A)** Total carbohydrate oxidation rates, **(B)** ingested carbohydrate oxidation rates and **(C)** endogenous carbohydrate oxidation rates **(C)** during SS. *Significant difference between GLU + MD and FRU + MD (*p* < 0.05).

Ingested and endogenous (i.e., carbohydrates stored in the body before the onset of carbohydrate ingestion during recovery) carbohydrate oxidation rates are presented in Figure 3. There was no difference in total carbohydrate oxidation rates between GLU + MD and FRU + MD (*p* = 0.080). There was only a main effect of time for overall carbohydrate oxidation (*p* < 0.001). Based on the findings that oxidation of ingested carbohydrates could only be accurately determined after ~21 min of exercise, statistical analysis for oxidation of ingested and endogenous carbohydrates has only been calculated from 30-min time point onwards. There was a time × condition interaction in ingested carbohydrate oxidation rates (*p* = 0.003) with higher oxidation rates of ingested carbohydrates at 30 and 45-min time points in FRU + MD as compared to GLU + MD (*p* < 0.001). Inversely, there was a trend toward higher endogenous carbohydrate oxidation rates in GLU + MD at 30-min time point during SS (time × condition interaction; *p* = 0.061) Furthermore, there was a tendency for higher fat oxidation rates in GLU + MD vs. FRU + MD (*p* = 0.054) and an overall main effect of time for fat oxidation (*p* < 0.001).

There was no difference in oxygen uptake ($\overset{∙}{\text{V}}$O_2_) (*p* = 0.157), RPE (*p* = 0.366) or HR (*p* = 0.570) between both conditions (Table 1). However, there was a main effect of time for RPE (*p* < 0.001).

**Table 1:** Oxygen uptake, fat oxidation rates, rate of perceived exertion and heart rate during the steady state part of exercise bout.

**Time (min)**	**Trial**	**$\overset{∙}{\text{V}}$O_**2**_ (L·min^**−1**^)**	**Fat (g·min^**−1**^)**	**RPE (6-20)**	**HR (bpm)**
15	GLU + MD	2.53 ± 0.26	0.25 ± 0.07	12 ± 1	137 ± 12
	FRU + MD	2.51 ± 0.27	0.20 ± 0.11	12 ± 1	137 ± 10
30	GLU + MD	2.56 ± 0.31	0.36 ± 0.12*	13 ± 2	138 ± 11
	FRU + MD	2.52 ± 0.27	0.25 ± 0.11*	13 ± 1	138 ± 11
45	GLU + MD	2.53 ± 0.26	0.50 ± 0.13*	13 ± 1*	139 ± 11
	FRU + MD	2.50 ± 0.30	0.36 ± 0.11*	13 ± 2*	135 ± 11
60	GLU + MD	2.63 ± 0.29	0.54 ± 0.12	14 ± 2	138 ± 10
	FRU + MD	2.51 ± 0.27	0.44 ± 0.12	14 ± 1	135 ± 12

* *Significantly different to a previous time point*.

### Blood Metabolites

**Figure 4:**
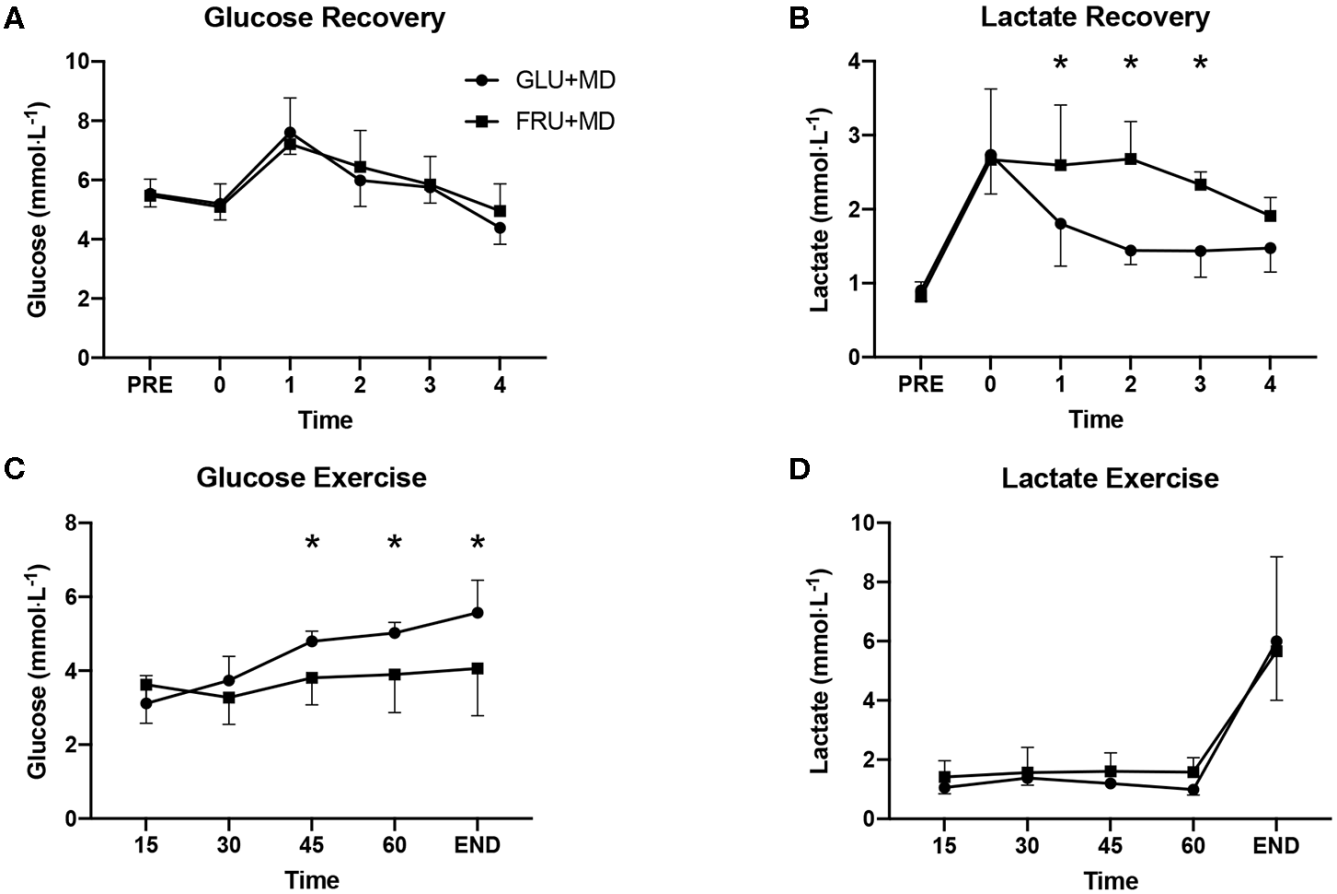
Plasma **(A)** glucose during the 4-h recovery period, **(B)** lactate during the 4-h recovery period, **(C)** glucose during the steady state exercise, and **(D)** lactate during steady state exercise. *Significant difference between GLU + MD and FRU + MD (*p* < 0.05).

Plasma metabolites (Figure 4) were analyzed separately for the recovery and subsequent exercise bout. Although care was taken to maintain patency of inserted cannulas, it was sometimes not possible to obtain blood samples from all participants. Thus, we were only able to analyse samples for five and six participants' during recovery and the subsequent exercise bout, respectively.

There were no differences in glucose concentrations between trials during recovery (*p* = 0.163), however there was a main effect of time (*p* < 0.001). For lactate concentrations there was a significant interaction between condition and time (*p* = 0.006) and *post-hoc* analysis showed significantly higher concentrations 1-h (*p* = 0.03), 2-h (*p* < 0.001), and 3-h (*p* = 0.01) into recovery in FRU + MD.

During the SS, there was a condition x time interaction for glucose (*p* < 0.001). Concentrations of plasma glucose were higher in GLU + MD at 45-min (*p* < 0.001), 60-min (*p* < 0.001) and at the end of the TT (*p* < 0.001), whereas there was only a significant effect of time for lactate (*p* < 0.001).

### GI Comfort

There was no difference in GI comfort between trials in any of the measures (*p* > 0.05). During recovery average values for nausea, stomach fullness and abdominal cramping were 1.0 ± 0.0 and 1.0 ± 0.0; 1.2 ± 0.4 and 1.0 ± 0.1; and 1.6 ± 1.6 and 1.9 ± 1.7 for GLU + MD and FRU + MD, respectively. Average values during exercise for nausea, stomach fullness and abdominal cramping being 1.3 ± 0.4 and 1.2 ± 0.4; 1.0 ± 0.1 and 1.1 ± 0.2; and 1.8 ±1.3 and 1.6 ± 1.0 for GLU + MD and FRU + MD, respectively.

## Discussion

The main aim of the present study was to assess whether ingestion of a combination of fructose and glucose as compared to glucose only during a 4-h recovery period after a glycogen reducing exercise protocol would improve subsequent pre-loaded cycle TT performance. Contrary to expectations, which were based on a previous observation of improved subsequent exercise capacity with post-exercise fructose-glucose vs. glucose only provision (14), there were no differences in performance outcomes between the two conditions. Accordingly, these data are discussed in the context of previous work and the metabolic and perceptual observations also made in the present study.

Maunder et al. (14) investigated effects of fructose and glucose based carbohydrates on exercise recovery and estimated oxidation rates of carbohydrates ingested in recovery during subsequent exercise. However, due to the duration of the subsequent exercise bout, comparisons between conditions were limited to the first 30 min of exercise. Most researchers report exogenous carbohydrate oxidations rates from 60 min of exercise onwards citing the potential to underestimate oxidation rates due to retention of ^13^C atoms within the body's bicarbonate pool (16). In the present study, breath ^13^CO_2_ recovery from ingested ^13^C labeled bicarbonate was determined as a proxy for the turnover of the bicarbonate pool and it was found that on average ~21 min were needed for the ingested ^13^C label to be eliminated. This observation is interpreted to mean that during moderate intensity exercise the aforementioned caution in reporting exogenous carbohydrate oxidation rates during the first 60 min of exercise is likely unnecessary. At least from a perspective of ^13^C retention within the body's bicarbonate pool, exogenous carbohydrate oxidation rates can be accurately determined from as early as 30 min after ^13^C labeled carbohydrate ingestion during exercise. This approach has been adopted in the present study and the finding will be useful for other researchers wishing to assess exogenous carbohydrate oxidation whilst employing protocols with a similar exercise intensity.

During SS higher oxidation rates of ingested carbohydrates in FRU + MD as compared to GLU + MD were observed from 30 to 45 min). Previous research would suggest the higher ingested oxidation rates were subsequent to higher carbohydrate availability in FRU+MD due to enhanced replenishment of liver (4, 10) and similar replenishment of muscle glycogen stores (12, 13). There was a trend toward higher endogenous carbohydrate oxidation rates in the first half of the SS in GLU + MD. The differential pattern in exogenous and endogenous carbohydrate oxidation in FRU-MD vs. GLU-MD converged to be similar by the end of the SS exercise bout, as was total CHO oxidation at that point. Furthermore, there was a clear trend for higher carbohydrate oxidation rates in FRU + MD and that might have offset the potential benefits of higher carbohydrate storage. In contrast, in the studies by Maunder et al. (14) and by Gray et al. (15), which used a slightly higher exercise intensity (70% $\overset{∙}{\text{V}}$O_2_max) than the present study, carbohydrate oxidation rates were comparable between both treatments. The extension in endurance running capacity with FRU + MD in the study by Maunder et al. (14) was temporally associated with higher total and ingested CHO oxidation at the point of fatigue in the GLU + MD condition, while this has not been observed by Gray et al. (15) Whilst it is not possible to say with certainty, it could be that at higher exercise intensities used by Maunder et al. (14) and Gray et al. (15) the larger storage of carbohydrates in FRU + MD enabled participants to exercise for longer.

As expected, during the recovery period plasma lactate concentrations were elevated in FRU + MD which is consistent with previous studies (4, 13–15), likely a result of fructose conversion into lactate by the liver (27, 28). Higher plasma lactate concentrations likely led to higher total carbohydrate oxidation rates during the recovery (not measured in present study) as observed previously (15, 29), which could contribute to lower net carbohydrate availability during the subsequent exercise bout in FRU + MD even if overall carbohydrate storage was higher. This difference in plasma lactate levels subsided by the end of recovery and there was no difference in lactate concentrations during the SS exercise. However, this does not necessarily mean that lactate flux did not continue to be elevated in FRU + MD, as increased flux could potentially explain higher oxidation rates of ingested carbohydrates during the SS exercise (30) especially considering the splanchnic-to-muscle lactate shuttle and given that lactate's preferential fate is oxidation (31). On the other hand, glucose concentrations did not differ during the recovery, remained stable on average during the SS in FRU + MD but were higher in the second part of the SS and at the end of TT in GLU + MD. A similar finding was observed previously (14) where glucose concentrations did not differ during the first 30-min of the exercise, but were higher in GLU + MD at the point of fatigue. That glucose concentrations in GLU + MD diverged from FRU + MD could have been a result of an ongoing absorption that is a consequence of a limited transport of glucose across the intestinal membrane (16) and whereas in FRU + MD absorption was complete during the recovery period, whilst it might not have been in GLU + MD. In the absence of performance differences between conditions, it thus seems unlikely that different plasma glucose concentrations influenced the performance outcomes. However, looking at the TT performance results, there appears to be an outlier whose performance was drastically worse in FRU+GLU as compared to GLU. This participant's plasma glucose concentrations reached a nadir of 2.1 mmol · L^−1^ at 30-min time point and stayed below 3 mmol·L^−1^ for the whole SS part of exercise. At the end of TT, concentration increased to 3.11 mmol·L^−1^. Low plasma glucose concentrations could have played a role in the performance outcomes of this participant, however, removing him from the results does not change the lack of significant differences in performance outcomes of the study.

Previous studies of carbohydrate feeding during exercise (32–34) have typically observed improved gut comfort with combined fructose and glucose feeding, possibly due to greater absorption than with glucose only feeding. Some observed performance benefits of fructose-glucose feeding during exercise have thus been attributed to improved gut comfort (35). Although suggested as a potential contributor toward improvements in exercise running capacity in FRU + MD in previous work (14), it has to be noted that the study did not find any statistically significant differences in GI comfort. The authors only speculated that this might contribute to overall findings due to an observed trend toward higher reported GI distress in GLU + MD condition. Reduced gut comfort would likely be more prevalent among runners than cyclists likely due to a higher mechanical stress as a result of horizontal movement of the body during running (36), and this has been documented (18). In the present study cycling was used as an exercise modality to try to minimize the effects that GI comfort might have on the study outcomes. In fact, the incidence of GI discomfort was practically non-existent in both conditions. This is in line with a recent work (15) that observed no differences in gastrointestinal discomfort. Collectively it seems that current evidence does not support the argument that short-term recovery of exercise performance/capacity is affected by GI comfort as a result of ingestion of different types of sugars.

This study is not without limitations. The menstrual cycle of the single woman participant was not controlled and given well documented effects menstrual cycle on metabolism, this could have influenced the results (37). However, it is important to note that neither her performance, substrate oxidation nor plasma metabolite responses were deviant from the rest of the sample. Nonetheless, caution is needed before generalizing the results of the present study to women athletes. Furthermore, it has to be acknowledged that plasma metabolite responses are not representative of the whole sample due to difficulties obtaining blood samples in three participants. However, close inspection of these participants' TT and substrate oxidation data reveals trends similar to those observed in other participants, which likely means that plasma metabolite responses would have been similar. In addition to that, lack of muscle and liver glycogen measurements present a limitation as well as assuming that protein oxidation during exercise was negligible.

## Conclusions

In summary, the present study demonstrates that ingestion of a combination of fructose and glucose as opposed to glucose only in recovery after an exhaustive exercise bout increases oxidation rates of ingested carbohydrates during subsequent exercise. However, in the conditions of the present study subsequent pre-loaded cycle time trial performance was not improved when fructose-glucose was provided during exercise recovery. Further research is required to better understand if and when combined ingestion of fructose and glucose during short term recovery from exhaustive exercise can improve subsequent exercise performance.

## Data Availability Statement

The datasets generated for this study are available on request to the corresponding author.

## Ethics Statement

The studies involving human participants were reviewed and approved by Science, Technology, Engineering and Mathematics Committee, University of Birmingham. The patients/participants provided their written informed consent to participate in this study.

## Author Contributions

TP and GW designed the study and interpreted the results and wrote the manuscript. TP recruited the participants, collected the data, carried out blood sample analysis, and performed the statistical analysis.

## Conflict of Interest

GW has received research funding and/or has acted as a consultant for GlaxoSmithKline Ltd., Sugar Nutrition UK, Lucozade Ribena Suntory Ltd, Dairy Management Inc. and Volac International Ltd. The remaining author declares that the research was conducted in the absence of any commercial or financial relationships that could be construed as a potential conflict of interest.
